# Physicians’ beliefs and attitudes about Benzodiazepines: a cross-sectional study

**DOI:** 10.1186/s12875-019-0965-0

**Published:** 2019-05-25

**Authors:** Inês Teixeira Neves, Joana Sara Silva Oliveira, Milene Catarina Coelho Fernandes, Osvaldo Rodrigues Santos, Vasco António Jesus Maria

**Affiliations:** 1 Instituto de Medicina Preventiva e Saúde Pública, Faculdade de Medicina, Universidade de Lisboa, Av. Prof. Egas Moniz, 1649-028 Lisbon, Portugal; 2 Administração Regional de Saúde de Lisboa e Vale do Tejo IP, Av. Estados Unidos da América 77, 1700-179 Lisbon, Portugal; 3 Instituto de Saúde Ambiental, Faculdade de Medicina, Universidade de Lisboa, Av. Prof. Egas Moniz, 1649-028 Lisbon, Portugal

**Keywords:** Benzodiazepines, Physicians, Beliefs, Attitudes, Survey

## Abstract

**Background:**

In 2015, Portugal was the OECD country with the highest reported consumption of BZD. Physician’s perceptions and attitudes regarding BZD are main determinants of related prescription habits. This study aimed to characterize beliefs and attitudes of Portuguese physicians regarding the prescription, management challenges, benefits, risks and withdrawal effects of BZD.

**Methods:**

A cross-sectional, observational study with online data collection through anonymous self-administered questionnaire. Physicians registered with the Portuguese Medical Association were invited to participate through direct e-mail message. Physicians were asked to give their opinion (using a 5-points Likert scale) regarding the prescription of BZD, their benefits and risks in the management of insomnia and anxiety, the possible adverse effects of chronic use and alternative non-pharmacologic approaches. Descriptive statistics were used and groups were compared through logistic regression.

**Results:**

A total of 329 physicians participated in the study (56% family physicians). Mean age was 44.10 ± 15.2 years, with 19.03 ± 14.9 years of clinical experience. Fifty eight percent of participants were female. Physicians reported BZD’s negative impact on cognitive function (89%), association with road traffic accidents (88%) and falls (79%). Also, 58% shared the belief that chronic use is justified if the patient feels better and without adverse events. Although 68% reported to feel capable of helping patients to reduce or stop BZD, 55% recognized difficulties in motivating them. Compared to other medical specialists (altogether), family physicians were significantly more aware about the adverse effects of BZD and considered that chronic use may not be justified. Conversely, more family physicians expressed concerns about their skills to motivate patients engaging in withdrawal programs and to support them during the process.

**Conclusion:**

Our results show that physicians’ awareness about risks of BZD chronic use is adequate though their attitudes and self-perceived skills towards promoting BZD withdrawal can be improved. Interventions in primary care are needed to capacitate physicians to better motivate patients for BZD withdrawal.

## Background

Benzodiazepines (BZD) were introduced in clinical practice as anxiolytic agents in the early 60s [1]. This class of drugs is used in the treatment of anxiety and sleep disorders, among other conditions for which their effectiveness is well established [2, 3]. Although BZD are approved for short-term treatment, its long-term use is common and recognized as a relevant public health problem [4, 5].

In the United States, a study reported that approximately 5.2% of adults (18–80 years) used BZD, and that the long-term consumption increased with age, from 14.7% (18–35 years) to 31.4% (65–80 years) [6]. Another study in British Columbia showed that between 1996 and 2006, the use of BZD increased from 7.8 to 8.4%, with 3.5% corresponding to long-term use in 2006 [7]. In Europe, the consumption of BZD is even higher. In Spain, BZD consumption has undergone continued growth since 2000, from a DHD (Defined Daily Dose per 1000 persons) of 56.7 to a DHD of 89.3 in 2012 [8, 9]. In Portugal, the consumption of BZD was 96 DHD in 2012, 6% more than in 2000 [10]. Compared with other European countries, Portugal is the OECD country with the highest reported consumption of BZD, with 114 DHD in 2015 versus 87 DHD in Spain and 16 DHD in the United Kingdom [11].

The BZD long-term use has been discouraged due to risks of dependence and negative impact on psychomotor abilities, particularly in older patients, with increased risk of falls and fractures [12] as well as road-traffic accidents [13]. More recently, BZD long-term use has been associated with cognitive deficits and dementia syndromes [14–17]. Hence, it is important to implement prevention programs for inappropriate BZD use and to motivate patients with chronic consumption to participate in supervised programs of BZD discontinuation [2, 18, 19].

Several studies regarding BZD prescription have shown that the decision to prescribe and maintain the BZD is complex and affected by several factors [20–23], including physicians’ knowledge, beliefs and attitudes regarding benefits and risks of BZD [24]. In fact, even though guidelines recommend that non-pharmacological interventions should be used as a first-line treatment for anxiety [25] and sleep disorders [26], the prescription of BZD may be perceived by medical doctors as an easier and more accessible way to address these health conditions [24], particularly in the primary healthcare setting. Indeed, though other medical specialists often decide BZD initiation, family physicians are the main prescribers in the long-term use [27, 28]. The limited consultation time per patient, the patients’ pressure for prescription refill and the perceived low risks and high effectiveness of BZD [24, 29] may influence whether or not to initiate, continue or terminate the prescription of BZD.

This study aimed to characterize beliefs and attitudes of Portuguese physicians regarding prescription, consumption and withdrawal of BZD. This is part of a broader project that evaluated the feasibility and effectiveness of a clinical protocol for BZD discontinuation at primary health care level.

## Methods

### Study design

A cross-sectional study was conducted with data collected from December 2015 to February 2016, among physicians registered with the Portuguese Medical Association (PMA). The study was approved by the Regional Health Administrations’ Ethics Committee and authorized by the Portuguese Data Protection Authority. Data were collected through Google Forms® and compiled into a protected database, only accessible to the research team.

### Instrument and procedure

An anonymous self-administered online questionnaire was developed for this study. The initial version of the questionnaire was developed taking into consideration results of qualitative studies investigating dimensions and factors related with BZD prescribing and usage [20–24, 29]. The questionnaire comprises four different sections: i) general beliefs about BZD, mainly about risks and benefits, ii) attitudes about prescription and chronic use of BZD, iii) self-perception of literacy about BZD and iv) self-efficacy perception for promoting withdrawal.

The questionnaire’s validation procedure consisted in discussions between the research team and two external experts on the relevance of the selected dimensions and factors (content validity). Additionally, six physicians of different specialties were invited to respond to the questionnaire to test general comprehension and consistency.

The final version of the questionnaire was named *Perception about Use of BZD Scale* (PUBS) and included 30 items (see Table 3). Respondents expressed their agreement with each of the statements through a 5-point Likert scale that ranged from “*1-Strongly disagree*” to “*5-Strongly agree*”. Physicians from all Specialist Colleges were invited to participate through direct e-mail, sent by the PMA. The email message included a brief information about the study and a link that landed in a webpage with more information about the participation procedure and goals. Physicians had access to the questionnaire after agreeing with the participation terms (by clicking on a “*I agree*” box). A reminder was sent by e-mail 1 month after the first message. Because answers were anonymous, the reminder was sent to the complete list of physicians, and it was requested to those that had participated in the first round to skip the invitation.

### Statistical analysis

Data were exported to and analyzed with IBM® SPSS® (version 22.0). Exploratory factor analysis was performed to study the structure of the questionnaire. The principal components extraction method with Varimax rotation was used. Each item was allocated to a factor when having a loading of 0.4 or higher. Sampling adequacy was tested with Kaiser–Meyer–Olkin (KMO) measure and sphericity was tested with Bartlett’s test. Cronbach’s *alpha* was used to assess the internal reliability of the complete set of items as well as for each dimension that emerged from the factor analysis.

The preliminary psychometric properties’ analysis fully supported the PUBS initial structure of four dimensions [30]. *1) Doctors’ beliefs about BZD* (items: 1, 2, 3, 4, 5, 6, 7, 8, 9, 10 e 11; alpha coefficient = 0.84); *2) Doctors’ attitudes about BZD prescription* (items: 13, 14, 15, 16, 17, 18, 19, 22, 23, 24, 26, 27, 30; alpha coefficient = 0.65); *3) Doctors’ self-perception of literacy about BZD* (items: 12, 21, 25; alpha coefficient = 0.60); *4) Doctors’ self-efficacy perception for promoting withdrawal* (items: 20, 28, 29; alpha coefficient = 0.41). The overall alpha coefficient of PUBS was 0.78.

The sample was compared with the population of physicians registered with the PMA regarding main sociodemographic variables and distribution by medical specialties. The data regarding the number of physicians registered with PMA was obtained through the official PMA website (www.ordemdosmedicos.pt).

Univariate descriptive analysis was conducted to describe PUBS items. Total score of the scale, as well as scores for each dimension, were computed as a linear sum. Logistic regression test was used for studying the association between type of medical specialty (family physicians versus other medical specialists, due to the small sample size of these other specialties) and agreement with each item. Comparisons of PUBS mean scores by type of medical specialty were done through Student’s *t* test for independent samples.

## Results

A total of 329 physicians, including 56% family physicians, participated in the study. The mean age was 44.1 ± 15.2 years, with 19.0 ± 14.9 years of clinical experience. Fifty eight percent of participants were female (Table 1). Regarding their clinical practice, 36% of the respondents worked mainly at a hospital. Regarding the category “other medical specialists”, 6% of respondents were psychiatrists with 20.4 ± 15.2 years of clinical experience and 5% were internists, with 17.4 ± 14.4 years of clinical experience.

**Table 1:** Characteristics of the respondents

	Family physicians (*n* = 184)	Other medical specialists (*n* = 145)	*p*-value	Total (*n* = 329)
Gender, n (%)				
Female	116 (63.0%)	74 (51.0%)	0.03^a)^	152 (57.7%)
Male	68 (37.0%)	71 (49.0%)		207 (42.3%)
Age group, *n* (%)*				
≤ 35 years	101 (55.2%)	50 (34.5%)	< 0.001^a)^	159 (44.4%)
36–55 years	27 (14.8%)	43 (29.7%)		81 (22.6%)
≥ 56 years	55 (30.1%)	52 (35.9%)		118 (33.0%)
Age, mean ± sd*	41.5 ± 14.3	46.7 ± 15.9	0.002^b)^	44.1 ± 15.2
Years of clinical practice, mean ± sd	16.2 ± 14.1	22.0 ± 15.5	< 0.001^b)^	19.0 ± 15.0
Nr of patient with prescribed BZD for the first time (last 3 months)				
No patients	31 (16.8%)	41 (28.3%)	< 0.001^a)^	76 (21.2%)
Between 1 and 5 patients	121 (65.8%)	55 (37.9%)		192 (53.5%)
Between 6 and 10 patients	20 (10.9%)	17 (11.7%)		39 (10.9%)
More than 10 patients	5 (2.7%)	16 (11.0%)		25 (7.0%)
Not applicable	7 (3.8%)	16 (11.0%)		27 (7.5%)

^a)^ Chi-square test for independence

^b)^ *Student’s t* test for independent samples

*One missing value for variable *Age* (*n* = 183)

Table 2 presents the answers to the PUBS questionnaire. Regarding beliefs about BZD, 30% of physicians considered that patients get a high-quality sleep with BZD, with significant differences between family physicians and other specialists. Most respondents were aware of BZD negative impact on cognitive function (89%) and about BZD association with road traffic accidents (88%) and falls (79%). Compared to other physicians, family physicians were significantly more aware of BZD impact on cognitive function (OR = 4.75), of BZD association with falls (OR = 5.48) and of increased risk of road traffic accidents (OR = 2.49).

**Table 2:** Responses to PUBS item: comparison between family physicians and other specialists

Items	Family physicians (*n* = 184)		Other specialists (*n* = 145)		*OR for agreement (95% CI)* ^*c)*^
n (%)	*Agreement* ^*a*^	*Disagreement* ^*b*^	*Agreement* ^*a*^	*Disagreement* ^*b*^	
Doctors’ beliefs about BZD					
1. With BZD, the patient gets a high-quality sleep	44 (23.9%)	94 (51.1%)	55 (37.9%)	57 (39.3%)	1.94** (1.21–3.13)
2. With BZD, the patient does not wake up so many times during night	115 (62.5%)	28 (15.2%)	93 (64.1%)	33 (22.8%)	1.07 (0.68–1.69)
3. With BZD, the patient feels more rested when waking up in the morning	50 (27.2%)	73 (39.7%)	39 (26.9%)	54 (37.2%)	0.99 (0.60–1.61)
4. With BZD, the patient feels less angry	99 (53.8%)	32 (17.4%)	96 (66.2%)	23 (15.9%)	1.68 * (1.07–2.64)
5. Chronic use of BZD does not represent a health risk to the patient	7 (3.8%)	170 (92.4%)	11 (7.6%)	119 (82.1%)	2.08 (0.78–5.49)
6. Chronic use of BZD contributes to the patients’ well-being	41 (22.3%)	81 (44%)	48 (33.1%)	48 (33.1%)	1.73* (1.06–2.82)
7. Chronic use of BZD is essential to patients’ anxiety control	51 (27.7%)	85 (46.2%)	48 (33.1%)	67 (46.2%)	1.29 (0.80–2.07)
8. Chronic use of BZD is a public health problem	157 (85.3%)	12 (6.5%)	113 (77.9%)	14 (9.7%)	1.62 (0.72–3.64)
9. Chronic use of BZD enhances the risk of several falls	161 (87.5%)	6 (3.3%)	98 (67.6%)	20 (13.8%)	5.48** (2.13–14.10)
10. Chronic use of BZD may impair cognitive performance	174 (94.6%)	4 (2.2%)	119 (82.1%)	13 (9%)	4.75** (1.51–14.92)
11. Chronic use of BZD increases the risk of road traffic accidents	168 (91.3%)	5 (2.7%)	121 (83.4%)	9 (6.2%)	2.49* (0.82–7.64)
Doctors’ attitudes about BZD prescription					
13. BZD consumption in unnecessary in most cases	121 (65.8%)	24 (13%)	83 (57.2%)	25 (17.2%)	1.52 (0.81–2.84)
14. It is important to inform the patient about the risk of tolerance associated with BZD	181 (98.4%)	1 (0.5%)	138 (95.2%)	1 (0.7%)	1.31 (0.08–21.16)
15. It is important to inform the patient about the risk of addiction associated with BZD	183 (99.5%)	–	138 (95.2%)	4 (2.8%)	0.10 (0.01–0.89)
16. Chronic use of BZD is justified if the patient feels better and without side effects	47 (25.5%)	94 (51.1%)	71 (49.0%)	44 (30.3%)	2.79** (1.76–4.45)
17. I feel pressured by patients to prescribe BZD	125 (67.9%)	33 (17.9%)	44 (30.3%)	74 (51%)	6.37** (3.73–10.88)
18. Patients feel like they are not taken seriously when I don’t prescribe BZD	49 (26.6%)	83 (45.1%)	20 (13.8%)	90 (62.1%)	2.66** (1.46–4.84)
19. When I refuse to prescribe BZD, I’m challenging the patient-doctor relationship	37 (20.1%)	98 (53.3%)	7 (4.8%)	109 (75.2%)	5.88** (2.51–13.79)
22. There is an acceptable level of anxiety and the doctor should help people to deal with it	173 (94.0%)	3 (1.6%)	140 (96.6%)	2 (1.4%)	0.82 (0.14–4.99)
23. The easiest way to deal with a patients’ anxiety is to prescribe a BZD	44 (23.9%)	123 (66.8%)	28 (19.3%)	106 (73.1%)	0.76 (0.45–1.29)
24. Prescribing BZD in clinical cases of anxiety is the most appropriate way to deal with those cases	14 (7.6%)	119 (64.7%)	16 (11.0%)	91 (62.8%)	1.51 (0.71–3.19)
26. Non-pharmacological approaches for anxiety need to be complemented with medication	39 (21.2%)	63 (34.2%)	32 (22.1%)	52 (35.9%)	1.05 (0.62–1.79)
27. Non-pharmacological approaches for sleep disorders need to be complemented with medication	39 (21.2%)	80 (43.5%)	39 (26.9%)	49 (33.8%)	1.37 (0.82–2.28)
30. Non-pharmacological approaches are appropriate for most patients	94 (51.1%)	45 (24.5%)	59 (40.7%)	37 (25.5%)	1.31 (0.76–2.26)
Doctors’ self-perception of literacy about BZD					
12. I consider myself well informed about the benefits and risks of BZD	161 (87.5%)	5 (2.7%)	94 (64.8%)	21 (14.5%)	0.26** (0.15–0.46)
21. I don’t feel capable of helping patients to stop/reduce the BZD consumption	19 (10.3%)	136 (73.9%)	21(14.5%)	90 (62.1%)	0.60 (0.31–1.18)
25. My knowledge on non-pharmacological approaches is enough to help patient not to choose for BZD	68 (37.0%)	64 (34.8%)	46 (31.7%)	61 (42.1%)	0.79 (0.50–1.27)
Doctors’ self-efficacy perception for promoting withdrawal					
20. I have difficulties in motivating patients to stop BZDs’ consumption	114 (62.0%)	48 (26.1%)	66 (45.5%)	40 (27.6%)	1.44** (0.86–2.42)
28. Psychological treatment of anxiety is of difficult access	147 (79.9%)	27 (14.7%)	104 (71.7%)	22 (15.2%)	0.64 (0.38–1.06)
29. It is difficult to motivate patients to see a psychologist	119 (64.7%)	38 (20.7%)	81 (55.9%)	36 (24.8%)	0.69 (0.44–1.08)

* Statistically significant (p-value < .05); ** Highly statistically significant (*p*-value < .01)

^a)^ Percentage of agreement (answers 4 and 5) ^b)^ Percentage of disagreement (answers 1 and 2). c) reference category: family physicians

Neutral point (answer 3 - neither agree or disagree) is not included in the table but was considered for the OR estimation

When considering attitudes towards BZD prescription, 42% of respondents disagreed with the statement that chronic use is justified if the patient feels better with no observed side effects. Although 68% felt capable of helping patients to reduce/stop benzodiazepines, 55% recognized difficulties in motivating them. Half of the physicians (51%) agreed that they feel pressured by patients to prescribe BZD. Compared to other physicians, family physicians have higher odds in believing that patients feel they are not being taken seriously if the BZD are not prescribed, and that they might be compromising the patient-doctor relationship when not fulfilling such expectation.

Regarding the four dimensions of the PUBS, significant differences were found between family physicians and other medical specialists (Table 3). Family physicians had less positive beliefs regarding BZD and less positive attitudes towards BZD prescribing (*p* < 0.001). On the other hand, family physicians’ self-perception of literacy about BZD was significantly higher (*p* = 0.004), except when compared with psychiatrists (M = 3.58 versus M = 4.10).

**Table 3:** PUBS scores by medical specialty

		Family physicians	Other medical specialists	Total	*p*-value^a^
Doctors’ beliefs about BZD	Mean (1 to 5 Likert scale)	2.35	2.61	2.47	< 0.001*
	Std. deviation	0.04	0.05	0.56	
Doctors’ attitudes about BZD prescription	Mean (1 to 5 Likert scale)	2.40	2.66	2.52	< 0.001*
	Std. deviation	0.03	0.03	0.38	
Doctors’ self-perception of literacy about BZD	Mean (1 to 5 Likert scale)	3.58	3.37	3.47	0.004*
	Std. deviation	0.04	0.06	0.67	
Doctors’ perception of self-efficacy for promoting withdrawal	Mean (1 to 5 Likert scale)	3.35	3.30	3.33	0.440
	Std. deviation	0.04	0.05	0.57	

* *Statistically significant (p-value < 0.05)*

^a)^ *Student’s t* test for independent samples (comparisons of mean PUBS scores by type of medical speciality)

## Discussion

This study aimed to characterize beliefs and attitudes of the Portuguese physicians regarding BZD prescription, consumption and withdrawal. A self-administered online scale was specifically developed for this purpose, the PUBS, with good psychometric properties when applied to medical doctors from different specialties (alpha coefficient of 0.78). The coefficient for some of the components of the scale (namely the one about Doctors’ perception of self-efficacy for promoting withdrawal) are rather low, which is expected due to the low number of items [31]. In any case, the items included in each dimension make sense from a conceptual point of view.

We observed that most physicians were aware of BZD negative impact on cognitive function and their association with falls and fractures and with road-traffic accidents. The scarcity of recent studies investigating physicians’ beliefs and attitudes towards BZD prescription makes difficult the external validation of our results [32]. Some older studies appear to show less negative attitudes regarding BZD prescription [21, 33]. Risk awareness was higher among family physicians for each of these types of adverse effects, as compared with other specialists (OR = 4.75, 5.48 and 2.49 respectively). Family physicians’ self-perception of literacy regarding BZD was also significantly higher when compared to other medical specialists (*p* = 0.004). Family physicians considered themselves well informed about benefits and risks of BZD (88%). However, regarding self-efficacy for promoting withdrawal, there were still 26% that didn’t feel capable to motivate patients to stop or reduce BZD consumption. Hence, being aware of the risks of BZD chronic use seems to be not enough to capacitate physicians in motivating patients to cease consumption. In fact, a systematic review published in 2013, that included studies conducted in Europe, United Sates, Australia and New Zealand, revealed that family physicians report challenges in helping patients to withdraw from BZD [29]. Since family physicians are usually responsible for prescription refills, the awareness about BZD risks shown in our study may promote and facilitate interventions aimed at reducing its inappropriate and chronic use.

Some studies have reported that physicians may feel ambivalent about the decision to prescribe and maintain the BZD consumption [21, 23, 29]. Concepts such as i) the role of health professionals in BZD prescription; ii) their perception about patients’ expectations in receiving a prescription during the appointment; iii) the desire to maintain good doctor-patient relationship; iv) and the difficulty in motivating the patient to accept non-pharmacological interventions, all these are central in BZD inappropriate prescribing decision [24, 27, 29, 34]. Our results are also consistent with these concepts, since we observed that family physicians have higher odds in believing that patients feel they are not being taken seriously when the physician refuses a BZD prescription refill, and that this might compromise the doctor-patient relationship. Also, half of the physicians (51%) reported that they feel pressured by patients to prescribe BZD and 55% recognized difficulties in motivation them to withdraw BZD. The long-term use of these drugs may also be influenced by other aspects such as: i) patients’ lack of knowledge about the risks and benefits of BZD [21]; ii) patients’ social or financial problems; iii) the medicalization phenomenon of human suffering and iv) shortage of timely available services of psychological counselling and brief psychotherapeutic interventions [27, 35, 36]. Furthermore, several studies support the hypothesis that BZD proper use depends on the information provided to patients by the prescriber [22, 24, 29].

Difficulties felt by family physicians could be explained by lack of training on how to manage beliefs, motivations and expectations of BZD chronic users, as well as lack of training for managing the BZD discontinuation from a pharmacological perspective. Though recommendations exist on the cautious prescription and utilization of BZD in the treatment of anxiety and insomnia, there are no formal guidelines in Portugal on how to proceed to help patients cease or reduce BZD consumption.

Therefore, we believe that there is a need to develop and validate a clinical protocol to support physicians and patients in the process of BZD withdrawal at Primary Care level. In addition, family physicians should receive adequate training in motivational competencies, in order to facilitate the patients’ adherence to the discontinuation program. Integrating motivational interview principles [37] with discontinuation programs may be a strategic and effective way-to-go, for handling resistant or ambivalent patients regarding BZD’s withdrawal [38].

Regarding factors influencing initiation and maintenance of BZD chronic use, 68% of family physicians considered themselves to be pressured by patients to prescribe BZD. This result is consistent with a study conducted by Anthierens and colleagues [21] in which family physicians reported that they were cautious in initiating BZD usage, but at the same time, felt overwhelmed by the psychosocial problems of their patients. This difficulty in dealing with patient’s complaints of anxiety and insomnia and the few available non-pharmacological alternatives at a short-term period, can easily lead to BZD over-prescription.

Some study limitations should be acknowledged. A total of 329 physicians responded to the questionnaire, which is a rather good sample. However an accurate calculation of the response rate was not possible. Even though it is known how many physicians are members of the Portuguese Medical Association, it was not possible to know how many of them actually received the online invitation to participate in the study. The percentage of physicians in our sample with 35 years or younger is 44.4%, which is lower than what is known for the population registered with the PMA [39]. Also, the use of an online approach could represent a bias towards younger physicians who are most prone to use the e-mail on a regular basis.

A reminder was sent 1 month after the initial e-mail message to increase the response rate. To ensure questionnaire anonymity, the reminder was sent to the complete list of physicians, requesting those that had participated in the first round to skip the invitation. However, the possibility of duplicate responses cannot be excluded with certainty.

It should be noted that this questionnaire was intended for all medical specialists. However, family physicians were the most represented specialists in the study. This may indicate that family physicians are more aware of the relevance of BZD utilization and consumption, since they are responsible for most BZD prescription refills.

Further studies to clarify the possible association between physicians and patients believes and attitudes towards BZD and the chronic use of these drugs are warranted.

## Conclusion

Our study showed that, in Portugal, physicians are aware of the risks of BZD chronic use. Family physicians seem to have significantly less positive beliefs and attitudes towards BZD’s prescription when compared with other specialists. They also have a better self-perception of literacy about BZD, although expressing concerns about their skills to help patients with BZD withdrawal. The results of this study represent a significant contribution to a better understanding of physicians’ beliefs and attitudes associated with prescription and continued use of BZD in Portugal. These results should be taken in consideration when addressing future medical educational interventions, especially aimed at capacitating physicians to motivate and help patients to cease BZD consumption.

## Abbreviations

BZD: Benzodiazepines
DHD: Defined daily dose per 1000 persons
KMO: Kaiser–Meyer–Olkin
OECD: Organization for Economic Co-operation and Development
PMA: Portuguese Medical Association
PUBS: Perception about Use of BZD Scale
